# Body Mass Index Combined With Inflammatory Factors Can Better Predict Varicocele

**DOI:** 10.7759/cureus.70072

**Published:** 2024-09-24

**Authors:** Si Yan Miao, Wen Rui Wu, Liang Feng, Qiang Chen

**Affiliations:** 1 Department of Urology, The First Affiliated Hospital of Nanchang University, Nanchang, CHN

**Keywords:** body mass index: bmi, inflammatory factors, male factor infertility, propensity score matching (psm), varicocele

## Abstract

Most patients with VC have no symptoms, so they are often discovered due to male infertility. Early identification of them is a matter of concern for clinicians. A retrospective analysis of clinical data from patients between January 1, 2021, and February 1, 2024, was conducted. Patients were divided into VC and non-VC groups. Propensity score matching (PSM) was performed at a ratio of 1:1, and two cohorts with homogeneous baseline status were selected. Multivariate binary logistic regression and receiver operating characteristic (ROC) curve were used to analyze independent risk factors and protective factors and to evaluate their diagnostic value individually and in combination. A p-value <0.05 was considered statistically significant. A total of 256 patients with similar clinical characteristics were further analyzed after PSM in a 1:1 ratio of the 423 patients included in the study.

The two groups had statistically significant differences in systemic immune-inflammation index (SII), neutrophil-lymphocyte ratio (NLR), platelet-lymphocyte ratio (PLR), and body mass index (BMI) (p<0.05). Multivariate binary logistic regression analysis showed that SII and NLR were independent risk factors for VC, while high BMI could reduce the prevalence of VC. The PLR differences were not significant. The ROC analysis showed that BMI, SII, and NLR could predict VC, with areas under the curve of 68.3% (cut-off value 22.32), 83.4% (cut-off value 357.57), and 83.2% (cut-off value 1.8), respectively. The combination of BMI and inflammatory factors was more accurate for predicting VC than BMI alone (87.5% vs. 68.3%, p=0.0001), SII (87.5% vs. 83.4%, p=0.0106), and NLR (87.5% vs 83.2%, p=0.0058).

Both SII and NLR are independent risk factors for VC while BMI is an independent protective factor. The BMI, SII, and NLR values have the potential to predict VC. The BMI combined with these inflammatory factors can improve the accuracy of prediction.

## Introduction

Varicocele (VC) is a common disease characterized by the curvature of venous plexuses in an earthworm-like shape, which can cause male infertility in severe cases. The vast majority of patients suffer from it on the left side, and nearly half of male infertility patients have VC, whether primary or secondary. Because VCs are one of the common causes of male infertility, early detection of this condition is essential. The common VC symptoms are discomfort or pain in the scrotum, which can worsen after prolonged standing or walking. The mechanism of its occurrence may be related to tissue ischemia and the traction of nerves by varicose veins. However, symptomatic cases account for only 2% to 10% of all cases of VC [1,2] and are often discovered unexpectedly or when seeking medical attention owing to male infertility.

Previous studies have shown that VC can cause complex pathophysiological changes in patients. Testicular temperature, cellular hypoxia, reactive oxygen species damage, microcirculatory dysfunction, and immunoglobulins and anti-sperm antibodies [3] suggest that patients with VC may have higher levels of inflammatory immune responses. However, there is currently no suitable indicator to reflect the inflammatory immune status of these patients. In addition, researchers have found that patients with higher body mass index (BMI) are less likely to develop VC, which may be related to reduced venous pressure. Yagmur found that the neutrophil-to-lymphocyte ratio (NLR) and platelet-to-lymphocyte ratio (PLR) were significantly elevated in the VC group compared to the control group [4]. Erdogan et al. discovered that VC patients with a lower systemic immune-inflammation index (SII) exhibit a higher surgical success rate [5]. Previous studies have suggested that blood parameters and BMI have potential predictive value for VC.

There are currently few studies on predicting VC, and blood parameters and BMI are easily obtained in outpatient and inpatient settings. Hence, we conducted a retrospective study to evaluate the value of blood parameters, both independently and in combination with BMI, in predicting the development of VC.

## Materials and methods

This is a retrospective analysis of clinical data from patients who underwent VC diagnostic procedures in our hospital's inpatient department and physical examination center from 2021 to 2024. The participants were divided into two groups based on the presence of VC. The diagnosis of VC was made according to the recommended diagnostic strategy in the guidelines, which includes evaluating whether the patient experiences pain or discomfort in the scrotum or groin area, conducting a physical examination to detect VC, and confirming the diagnosis with color ultrasound imaging. Patients who did not meet the diagnostic criteria were classified as the non-VC group. The Ethics Committee of The First Affiliated Hospital of Nanchang University approved all our study procedures (approval no. IIT/2024/192).

Inclusion and exclusion criteria

Patients under 45 years old with no pneumonia, urinary tract infection, malignant tumor, diabetes, hypertension, or fever caused by other diseases were included in the study. Those over 45 years old, having urinary tract infections, pneumonia, malignant tumors, hypertension, diabetes, or fever caused by other diseases were excluded from the study.

Data collection

Before breakfast, blood samples were collected from patients in a fasting state. The samples underwent testing for a complete blood count, fasting blood glucose levels, cholesterol levels, triglycerides, and liver and kidney function tests using automated analyzers. All inflammatory factors were automatically calculated by a computer using a recognized formula. An electronic medical record system at the hospital collected the above information. As for how to confirm that the blood draw was performed in a fasting state, we verified the timing of the blood draw and the patient's condition by reviewing the nursing records. Relevant indicators were calculated: BMI as weight(kg)/height2(m) and SII as neutrophil count(X109/L) × platelet count(X109/L)/lymphocyte count(X109/L); PLR as platelet count(X109/L)/lymphocyte count(X109/L) and NLR as neutrophil count(X109/L)/lymphocyte count(X109/L); platelet-albumin ratio (PAR) as platelet count(X109/L)/albumin value(g/L).

Data analysis

Statistical analysis was completed using SPSS Statistics version 27 (IBM Corp., Armonk, NY, USA) and MedCalc version 22 (MedCalc Software Ltd., Ostend, WV, BEL). Continuous variables were expressed as the mean ± SD for those following a normal distribution and as the median (interquartile range) for variables with a non-normal distribution. Categorical variables were expressed as percentages. For the analysis of continuous variables, the independent sample t-test was utilized for variables with a normal distribution between two groups, and the Mann-Whitney U test was used for those with a non-normal distribution. The Pearson chi-square test was used to analyze categorical variables. Binary logistic regression analysis was used to identify potentially essential predictors and control confounding variables. Based on receiver operating characteristic (ROC) analysis, we validated diagnostic efficacy and calculated Youden's index for optimal diagnostic value. The ROC-Delong tests were used to compare two factors. A two-sided p-value of 0.05 was considered statistically significant.

Due to the differences in baseline data between patients, to maintain consistency between the two groups of patients and avoid selection bias, we used 1:1 propensity score matching (PSM) to make the baseline data of the two groups consistent. We included age, smoking, alanine aminotransferase, aspartate aminotransferase, total protein, globulin, creatinine, urea, uric acid, glucose, triglycerides, and cholesterol into the matching, with a caliper value of 0.02.

## Results

**Figure 1:**
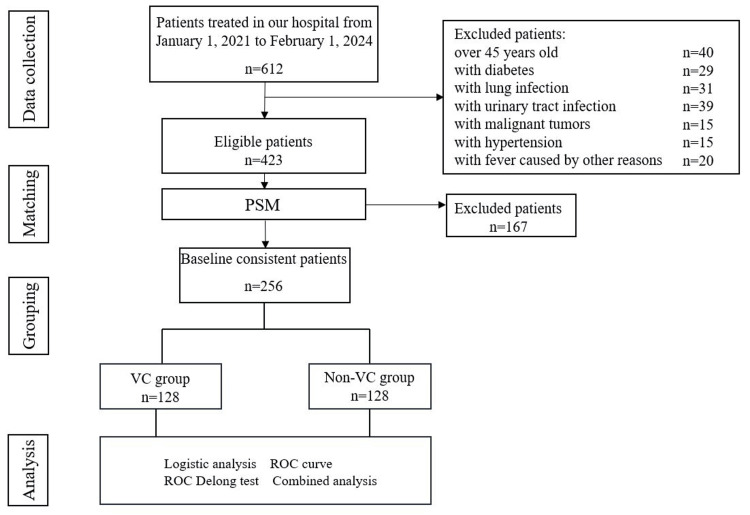
Research flow chart VC: Varicocele

We reviewed 612 clinical data of patients who visited our hospital from January 1, 2021, to February 1, 2024. Among them, 40 patients were over 45 years old, 29 patients had diabetes, 31 patients had lung infections, 39 patients had urinary tract infections caused by stones, 15 patients had malignant tumors, 15 patients had hypertension, and 20 patients had fever caused by other reasons. Therefore, the remaining 423 patients were analyzed further. We conducted a 1:1 PSM on the remaining patients and ultimately included 256 patients with consistent baseline data for analysis. The study roadmap is detailed in Figure 1. The baseline data comparison between the non-VC group (n=226) and the VC group (n=197) before PSM showed differences in some indicators of liver and kidney function and blood lipids (p<0.05). After PSM, these differences were well eliminated. In Table 1, we present the baseline data of the two groups before and after PSM.

**Table 1:** Comparison of clinical baseline data between patients with and without VC VC: Varicocele; ALT: Alanine aminotransferase; AST: Aspartate aminotransferase; TP: Total protein; ALB: Albumin; GLB: Globulin; CRE: Creatinine; UA: Uric acid; TG: Triglyceride; CHO: Cholesterol; GLU: Glucose

Variables		Before matching			After matching	
	Non-VC (n=226)	VC (n=197)	p-value	Non-VC (n=128)	VC (n=128)	p-value
Age (years)	21 (18~26)	21 (19~26)	0.993	21 (17.3-26.0)	21 (19.0-25.0)	0.563
Smoke	43 (19.5%)	24 (12.2%)	0.057	19 (8%)	17 (7%)	0.719
ALT	23.1 (16.0~37.0)	16.2 (11.6~23.6)	<0.001	21.0 (14.0-30.0)	19.0 (12.3-30.5)	0.280
AST	22.0 (18.1~27.0)	18.7 (15.7~22.0)	<0.001	21.0 (17.0-24.8)	20.0 (17.2-24.5)	0.361
TP	68.8±6.1	70.0±5.8	0.032	69.8±5.9	69.1±5.5	0.356
ALB	43.8±3.9	44.8±3.9	0.009	44.2±4.0	44.2±3.8	0.936
GLB	24.6±3.4	25.2±3.2	0.088	25.1±3.3	24.9±3.2	0.590
CRE	78.5±15.8	79.2±11.5	0.569	78.1±15.3	79.1±11.6	0.564
Urea	4.9 (4.0~5.6)	4.8 (3.9~5.7)	0.254	4.7±1.2	4.9±1.2	0.262
UA	410.2±99.2	382.2±70.4	<0.001	394.3±98.8	393.1±75.7	0.910
GLU	4.5 (4.2~5.0)	4.6 (4.2~5.0)	0.831	4.5 (4.2-5.0)	4.6 (4.2-5.0)	0.748
TG	1.1 (0.8~1.7)	0.9 (0.7~1.3)	<0.001	1.0 (0.7-1.3)	0.9 (0.7-1.3)	0.643
CHO	4.2 (3.8~5.0)	3.8 (3.4~4.5)	<0.001	4.1±0.8	4.1±0.8	0.374

**Figure 2:**
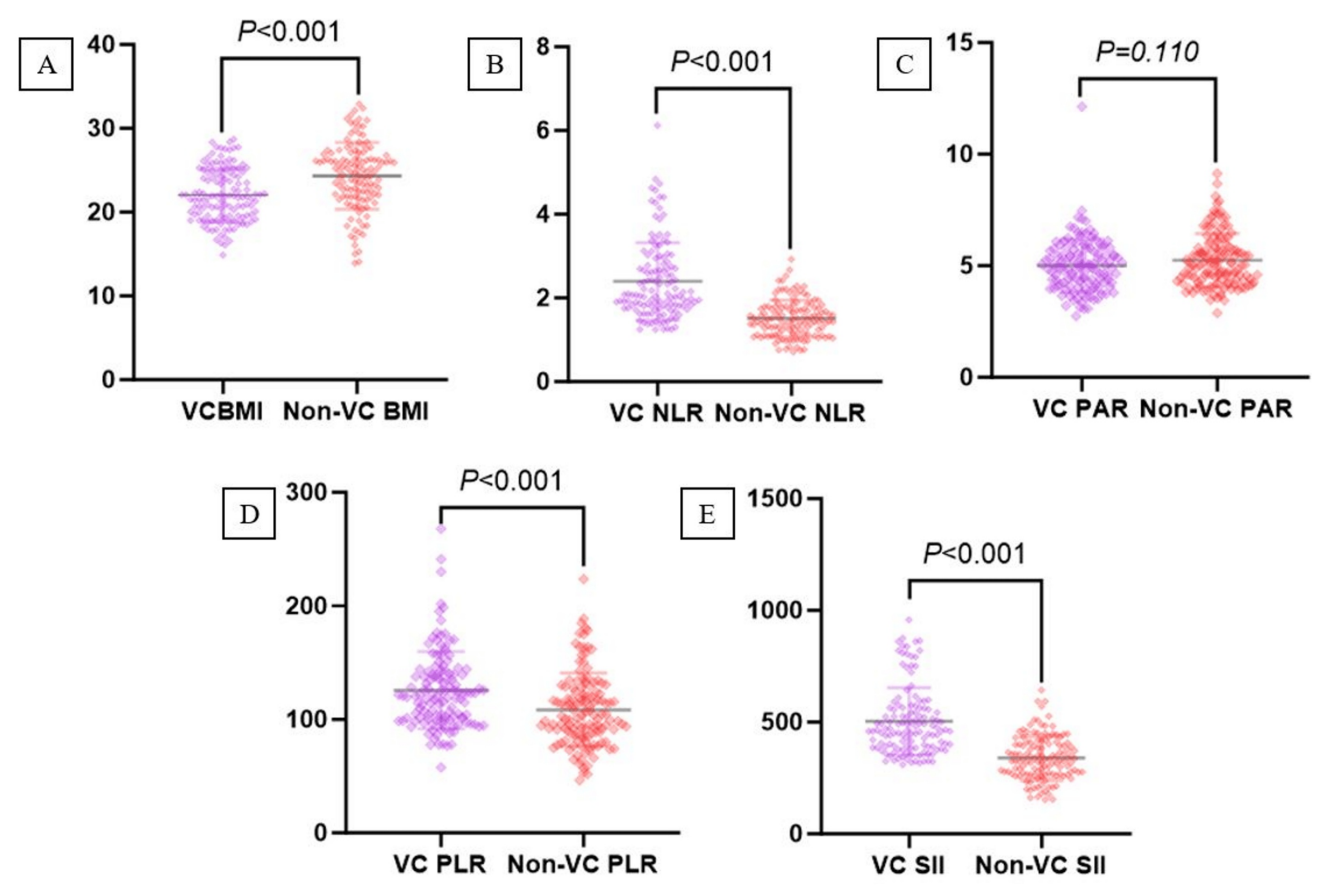
Comparison of BMI and inflammatory factors between VC and non-VC patients VC: Varicocele; NLR: Neutrophil-lymphocyte ratio; PAR: Platelet-albumin ratio; PLR: Platelet lymphocyte ratio; SII: Systemic immune-inflammation index

After the baseline data was balanced, a difference analysis was conducted on inflammatory factors and BMI. The BMI level of patients with VC was lower, while the NLR, SII, and PLR levels were higher. No difference was observed in PAR (Figure 2). On this basis, a logistic regression analysis was conducted on the indicators. The single-factor logistic regression analysis (Table 2) suggests that NLR, SII, and PLR increase the risk of VC, while BMI decreases the prevalence of VC. However, there may be confounding factors among them. Further multiple regression (Table 2) analysis showed that the OR value of NLR was 5.81 (95% CI: 2.24-15.04, P<0.001), the OR value of SII was 1.01 (95% CI: 1.00-1.01, p=0.009), and the OR value of BMI was 0.84 (95% CI: 0.77-0.92, p<0.001). However, PLR showed no statistical difference in the multiple regression analysis (p=0.62).

**Table 2:** Univariate and multivariate binary logistic regression analysis of the VC group and non-VC group VC: Varicocele; OR: Odds ratio, CI: Confidence interval

Variables			Univariate					Multivariable			
	β	SE	Z	p-value	OR (95%CI)	β	SE	Z	p-value	OR (95%CI)	
					1.00 (reference)					1.00 (reference)	
BMI	-0.18	0.04	21.86	<0.00	0.838 (0.778-0.902)	-0.17	0.05	13.81	<0.00	0.841 (0.768-0.921)	
					1.00 (reference)					1.00 (reference)	
NLR	2.54	0.36	49.71	<0.00	12.681 (6.259-25.693)	1.76	0.49	13.15	<0.00	5.810 (2.224-15.042)	
PLR	0.02	0.00	14.93	<0.00	1.016 (1.008-1.025)	0.00	0.01	0.25	0.62	1.003 (0.991-1.015)	
SII	0.01	0.00	52.02	<0.00	1.013 (1.010-1.017)	0.01	0.00	6.78	0.01	1.007 (1.002-1.012)	

**Figure 3:**
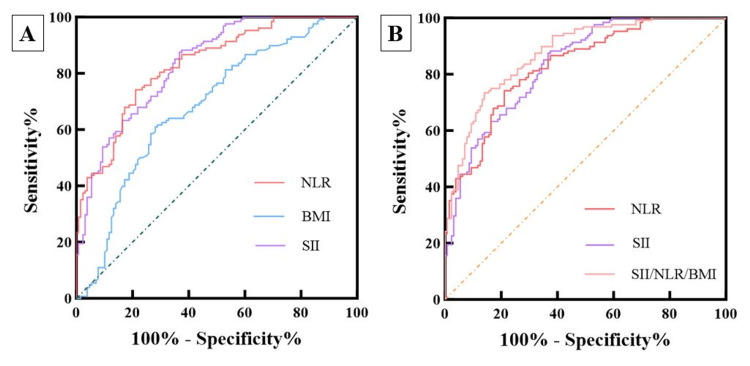
The predictive value of BMI and inflammatory factors for VC VC: Varicocele

The ROC analysis of variables with statistical differences suggested by multivariate logistic regression (Figure 3 A) showed that the areas under the ROC curve of NLR and SII were 83.2% and 83.4%, respectively. In comparison, the area under the curve for BMI was 68.3%. Their optimal cutoff values were 22.32 (BMI: sensitivity 60.9%, specificity 71.9%), 357.57 (SII: sensitivity 87.5%, specificity 63.3%), and 1.80 (NLR: sensitivity 74.2%, specificity 78.9%), respectively. The ROC-Delong test showed no significant difference in area under the curve (AUC) between SII and NLR (p=0.898). Both SII and BMI, as well as NLR and BMI, showed significant differences (p<0.05). For PLR, it showed no statistical difference in multi-factor binary logistic regression and was excluded as a confounding factor.

The BMI, SII, and NLR were combined using logistic regression and subjected to ROC analysis (Figure 3 B). The results showed that the area under the ROC curve for the combined indicators was 87.5%. The comparison of the combined indicators showed that they were superior to the individual indicators (87.5% vs. 83.4%, p=0.0106; 87.5% vs. 83.2%, p=0.0058; 87.5% vs. 68.3%, p=0.0001). The sensitivity and specificity were 73.4% and 85.9%, respectively, and the accuracy and balance were the best.

## Discussion

Patients with VC often have no symptoms until male infertility issues arise [6], emphasizing the importance of identifying new indicators for high-risk individuals. Our study introduces such indicators, offering a novel approach for screening VC. The mechanism of VC leading to male infertility is complex, yet its pathological and physiological changes are still under investigation. Evidence from both animal experiments [7] and real-world patients [8,9] has shown that in this condition, pro-inflammatory cytokines are abnormally elevated, and inflammatory pathways are activated [10]. Micheli et al. found that sperm apoptosis in patients with leukocytospermia and VC was significantly increased compared to the control group. Sperm apoptosis and necrosis were positively correlated with tumor necrosis factor-alpha (TNF-α), interleukin-6 (IL-6) levels, malondialdehyde (MDA) concentration, and catalase activity, while negatively correlated with ghrelin and obestatin [11]. Some pharmacological studies have also found evidence of downregulating inflammation in patients with VC through drugs [12-14]. Hassan et al. studied the effect of berberine on VC rats, and the results showed that berberine significantly reduced the levels of IL-6, TNF-α, nitric oxide (NO), and malondialdehyde (p<0.05). These studies primarily focus on interleukin and tumor necrosis factor.

This study shows that SII and NLR are risk factors for VC. The SII is a new biomarker widely studied by scholars in many different specialties and has achieved certain results [15]. Recent studies suggest that SII can be used to evaluate the prognosis of cancer patients and can increase the risk of diabetes-related complications [16], peripheral vascular diseases [17], ulcerative colitis [18], and other diseases. Other studies have shown that SII is related to diabetes [19], hypertension [20], urinary tract infections [21], and malignant tumors [22]. The NLR is an easily accessible marker widely used in almost all medical disciplines because, whether infection is present or not, it can reliably reflect the immune response to various stimuli. The normal value of NLR is generally 1-2 [23]. To our knowledge, this is the first study to investigate the predictive significance of both factors for VC. Our research results show that an SII value of ≥357.57 or an NLR value of ≥1.8 significantly increases the probability of developing VC.

Some researchers found that the incidence rate of spermatic vein varicosities in obese patients was lower than that in patients with a leaner body type [24,25], potentially owing to the protective cushioning of abdominal fat against venous compression, similar to the nutcracker syndrome [26,27]. These studies and viewpoints suggest that among asymptomatic patients, those with leaner body types should be vigilant about screening for the presence of VC and nutcracker syndrome. Our research results are consistent with these findings. After reciprocal correction, while BMI also has a certain diagnostic efficacy, it is not as good as that of SII and NLR. There is currently little research on whether BMI and blood parameters differ among patients with different degrees of VC severity. Chen et al. studied the correlation between BMI, blood parameters, and the severity of VC. They did not observe stratified BMI and blood parameters differences among patients with different grades of VC. Body mass index and blood parameters alone cannot distinguish patients with varying grades of VC [28]. Varicocele does not have symptoms. The severity of the condition is not only related to ultrasound classification and physical examination classification but also to the duration of the disease. However, it is difficult to assess for asymptomatic patients.

In addition, we further combined the risk factors and protective factors obtained from this study for further analysis. This idea is based on the concept of combining multiple methods for disease diagnosis, such as prostate-specific antigen combined with nuclear magnetic resonance for prostate cancer diagnosis. Many scholars have applied similar methods of combining numerous indicators to increase differences and have achieved certain results. Zhou et al. combined the RDW with the bedside index of severity of acute pancreatitis (BISAP), and the results showed that the predictive effect was superior to that of RDW alone (0.787 vs. 0.872) [29]. Koezuka et al. combined the average and maximum CT values to improve the prediction accuracy of multiple squamous lesions in small lung adenocarcinoma with solid nodules (0.862 vs. 0.868) [30]. We also explored the value of this idea in predicting VC. Our research shows that BMI, SII, and NLR all have predictive values for VC, but SII and NLR have low specificity. Therefore, we combined BMI with them for analysis. The specificity of the combined prediction is the highest. Overall, low BMI serves as one of the causes of secondary VC and possesses certain predictive value. Meanwhile, inflammatory factors, as the consequences of VC, can cover both primary and secondary VC. Combining the two can yield better results.

Compared to other potential predictive factors such as tumor necrosis factor and interleukin, our indicator is relatively easier to obtain and less expensive, imposing a smaller economic burden on patients and facilitating clinical detection. Our research findings also contribute to exploring various treatment methods for VC. In terms of diagnosis, we should pay attention to VC screening among individuals with a low BMI. Regarding treatment, for patients with a low BMI, increasing BMI may alleviate the severity of VC. For inflammatory factors, anti-inflammatory drugs can be administered to mitigate the impact of VC on the testes rather than solely relying on surgical intervention.

This study has some shortcomings and limitations. The data in this study are all from a single center. Since most patients with VC have no symptoms, it was difficult to assess the relationship between the duration of the disease and SII or NLR. The relationship between SII, NLR, and spermatic vein semen quality needs further study. Due to the lack of records regarding patients' participation in sports activities and dietary habits, our research is incomplete in this regard, and we are unable to provide relevant tips and guidance to patients. Body mass index does not reflect the distribution of body fat and cannot fully explain all cases of VC. Our study excluded some patients, but in the real clinical setting, the patient population is often not so standardized, and they may have hypertension and diabetes, or they may be older. These limitations may restrict the applicability of the research results to a broader population. Additionally, this result requires verification through prospective studies, such as potential future prospective cohort studies, to observe whether there are differences in the incidence rate among patients with different BMIs. Observe whether the levels of inflammatory factors increase with the progression of the disease in VC patients and non-VC patients.

## Conclusions

The SII and NLR are independent risk factors for VC, and BMI is an independent protective factor. The former represents the effect, while the latter is the cause. The BMI, SII, and NLR can predict VC. The accuracy of the prediction is improved when the BMI is combined with these inflammatory factors. The relationship between inflammatory factors and the pathogenesis of VC and whether inflammatory factors can predict semen quality is unclear. Limited by a retrospective, single-center design, the results of this study need to be further confirmed by prospective studies.
